# Reduced accuracy of MRI deep grey matter segmentation in multiple sclerosis: an evaluation of four automated methods against manual reference segmentations in a multi-center cohort

**DOI:** 10.1007/s00415-020-10023-1

**Published:** 2020-07-03

**Authors:** Alexandra de Sitter, Tom Verhoeven, Jessica Burggraaff, Yaou Liu, Jorge Simoes, Serena Ruggieri, Miklos Palotai, Iman Brouwer, Adriaan Versteeg, Viktor Wottschel, Stefan Ropele, Mara A. Rocca, Claudio Gasperini, Antonio Gallo, Marios C. Yiannakas, Alex Rovira, Christian Enzinger, Massimo Filippi, Nicola De Stefano, Ludwig Kappos, Jette L. Frederiksen, Bernard M. J. Uitdehaag, Frederik Barkhof, Charles R. G. Guttmann, Hugo Vrenken, the MAGNIMS Study Group

**Affiliations:** 1 Department of Radiology and Nuclear Medicine, MS Center Amsterdam, Amsterdam Neuroscience, Amsterdam UMC, Location VUmc, De Boelelaan 1117, 1081 HV Amsterdam, The Netherlands; 2 Department of Neurology, MS Center Amsterdam, Amsterdam Neuroscience, Amsterdam UMC, Location VUmc, Amsterdam, The Netherlands; 3 Department of Human Neurosciences, “Sapienza” University of Rome, Rome, Italy; 4 Department of Neurosciences, San Camillo Forlanini Hospital, Rome, Italy; 5 Center for Neurological Imaging, Department of Radiology, Brigham and Women’s Hospital, Harvard Medical School, Boston, MA USA; 6 Department of Neurology, Medical University of Graz, Graz, Austria; 7 Neuroimaging Research Unit, Division of Neuroscience, Institute of Experimental Neurology, Milan, Italy; 8 Neurology Unit, IRCCS San Raffaele Scientific Institute, Milan, Italy; 9 Division of Neurology and MRI Research Center, Department of Medical, Surgical, Neurologic, Metabolic and Aging Sciences, University of Campania “Luigi Vanvitelli”, Naples, Italy; 10 Research Unit, Queen Square MS Centre, Department of Neuroinflammation, UCL Queen Square Institute of Neurology, University College London, London, UK; 11 Unitat de Ressonància Magnètica (Servei de Radiologia), Hospital Universitari Vall D’Hebron, Autonomous University of Barcelona, Barcelona, Spain; 12 Division of Neuroradiology, Vascular and Interventional Radiology, Department of Radiology Medical, University of Graz, Graz, Austria; 13 Neurophysiology Unit, IRCCS San Raffaele Scientific Institute, Milan, Italy; 14 Vita-Salute San Raffaele University, Milan, Italy; 15 Department of Neurological and Behavioural Sciences, University of Siena, Siena, Italy; 16 Department of Neurology, University Hospital, Kantonsspital, Basel, Switzerland; 17 Department of Neurology, Glostrup University Hospital Copenhagen, Copenhagen, Denmark; 18 Institutes of Neurology and Healthcare Engineering, UCL London, London, UK

**Keywords:** Multiple sclerosis, Deep grey matter, Atrophy, Automated segmentation methods

## Abstract

**Background:**

Deep grey matter (DGM) atrophy in multiple sclerosis (MS) and its relation to cognitive and clinical decline requires accurate measurements. MS pathology may deteriorate the performance of automated segmentation methods. Accuracy of DGM segmentation methods is compared between MS and controls, and the relation of performance with lesions and atrophy is studied.

**Methods:**

On images of 21 MS subjects and 11 controls, three raters manually outlined caudate nucleus, putamen and thalamus; outlines were combined by majority voting. FSL-FIRST, FreeSurfer, Geodesic Information Flow and volBrain were evaluated. Performance was evaluated volumetrically (intra-class correlation coefficient (ICC)) and spatially (Dice similarity coefficient (DSC)). Spearman's correlations of DSC with global and local lesion volume, structure of interest volume (ROIV), and normalized brain volume (NBV) were assessed.

**Results:**

ICC with manual volumes was mostly good and spatial agreement was high. MS exhibited significantly lower DSC than controls for thalamus and putamen. For some combinations of structure and method, DSC correlated negatively with lesion volume or positively with NBV or ROIV. Lesion-filling did not substantially change segmentations.

**Conclusions:**

Automated methods have impaired performance in patients. Performance generally deteriorated with higher lesion volume and lower NBV and ROIV, suggesting that these may contribute to the impaired performance.

**Electronic supplementary material:**

The online version of this article (10.1007/s00415-020-10023-1) contains supplementary material, which is available to authorized users.

## Introduction

In multiple sclerosis (MS), atrophy of deep grey matter (DGM) structures like the caudate nucleus (caudate), putamen and thalamus is associated with cognitive and clinical impairment [1–4]. Accurate segmentations of these structures from structural MRI are key to understanding these atrophic processes and their role in MS.

However, it is unclear whether DGM segmentation using state-of-the-art automated methods is as accurate in MS cases as in healthy controls. Since studies have shown that white matter (WM) lesions and atrophy could affect measures such as whole-brain grey matter (GM) volume, it could be expected that such pathology also affects DGM segmentation [1, 5–9].

A direct comparison of automated methods to expert manual (reference) segmentation was performed by Derakhshan et al. (2010) in a small dataset containing 3 slices each of 3 MS patients [1]. Although that paper provided insights into the spatial overlap between automated and manual segmentations, with 3 slices per subject no volumetric analysis was possible. Moreover, the small number of subjects did not allow any analysis of relations between segmentation performance and MS-related pathological changes.

Therefore, this study quantitatively investigated automated segmentation performance in a whole-brain dataset of 32 subjects including MS patients and healthy controls. Four publicly available segmentation method packages (FSL-FIRST [10], FreeSurfer [11], Geodesic Information Flow (GIF) [12] and volBrain [13]) were evaluated in terms of volumetric and spatial agreement with manual segmentations created by combining manual outlines of three trained raters by majority voting. Moreover, the relation of segmentation accuracy with total and regional lesion load, whole-brain volume, and volume of the structure of interest was assessed to determine possible confounding disease relations factors.

## Methods

### Subjects

In total 21 MS patients and 11 healthy controls subjects were retrospectively selected from two of the multi-center studies of the MAGNIMS Study Group (www.magnims.eu) [14, 15]. Demographic details of the subjects are listed in Table 1. Subjects had been recruited at nine European centers, see Supplementary data A for the centers.

**Table 1:** Demographics of the subjects

Disease status	Number of cases (male/female)	Average age in years ± std	Median EDSS score (range)	Average DD year ± std
HC	11 (3/8)	37.6 ± 8.2	n.a	n.a
MS	21 (9/12)	43.2 ± 10.1	3.5 (6.0)	9.5 ± 6.9
RRMS	10 (4/6	39.8 ± 8.3	2.3 (2.5)	8.0 ± 9.8
SPMS	5 (3/2)	41.3 ± 8.9	4.0 (6.0)	11.0 ± 5.3
PPMS	6 (2/4)	49.4 ± 10.8	3.5 (4.5)	14.0 ± 4.0

*EDSS* expended disability status scale, *DD* disease duration, *std* Standard deviation, *HC* healthy control, *RR* relapsing remitting, *SP* secondary progressive, *PP* primary progressive

The selection of the cases was based on maximizing the number of scanners and the number of secondary progressive MS (SPMS) and primary progressive MS (PPMS) cases while considering the workload for the three raters. All patients and controls had given informed consent for the use of their brain MRI-scans for research within the original study.

### Acquisition

An overview of acquisition parameters for each site is given in Supplementary Tables 1 and 2. Briefly, MRI data were obtained using magnets operating at 3 T for all cases with three vendors (Siemens, Philips and GE). One of the two following imaging protocols was used: (1) 3D T1‐weighted scan (different pulse sequences for different venders) and a dual-echo spin echo scan with both 2D T2-weighted and 2D proton density (PD) weighted; or (2) 3D T1-weighted magnetization prepared rapid gradient echo (MPRAGE) scan and 2D fluid-attenuated inversion recovery (FLAIR) T2-weighted fast spin-echo sequence.

### Manual segmentation of DGM structures

Manual segmentation of three DGM structures was performed using the SPINE online environment for collaborative research (https://spinevirtuallab.org). The caudate nucleus, putamen and thalamus were all manually segmented on the full 3D T1-weighted images in each subject by each rater. Four scans were outlined a second time by each rater to examine intra-rater variability. A summary of the segmentation protocol is added to the supplementary data (Supplementary Protocol 1).

The manual segmentations of the three raters were combined into a reference using majority voting: i.e., a voxel was classified as part of a structure if at least 2 of the 3 raters assigned it to that structure.

### Lesion segmentation

Lesion segmentation was also performed manually by one expert rater, on the FLAIR scan or on the PD scan. The lesion segmentation was performed using the Medical Image Processing, Analysis, and Visualization (MIPAV) software environment whereby only lesions of at least three voxels were included.

### Lesion filling

Lesion-filling is a common pre-processing step in patient scans, in which the intensities of voxels identified as being part of WM lesions are replaced by intensities similar to normal-appearing white matter. In this study, lesion-filling was applied using two algorithms: lesion segmentation toolbox (LST-LF) [16], and LEAP [8], and both versions of lesion-filled images as well as native images were analyzed. The lesion masks were first co-registered from their original PD or FLAIR space to 3D-T1 space using FSL-FLIRT with tri-linear interpolation and a threshold of 0.5 because this was previously found to provide good results for whole-brain GM volume measurements [6]. The Supplementary data B provides a description of LST-LF and LEAP. In this study, lesion-filling was applied using two algorithms: lesion segmentation toolbox (LST-LF) [16], and LEAP [8], and both versions of lesion-filled images as well as native images were analyzed. The lesion masks were first co-registered from their original PD or FLAIR space to 3D-T1 space using FSL-FLIRT with tri-linear interpolation and a threshold of 0.5, based on literature [6]. Second, the lesion was filled on the 3D-T1 weighted image with the use of the lesion mask in 3D-T1 space. The Supplementary data B provides a description of the two used lesion filling methods (LST-LF and LEAP).

### Automatic DGM segmentation method

Within this study four automatic DGM segmentation methods were assessed; FSL-FIRST (https://fsl.fmrib.ox.ac.uk/fsl/fslwiki/FIRST), FreeSurfer (https://surfer.nmr.mgh.harvard.edu), volBrain (https://volBrain.upv.es) and GIF (https://niftyweb.cs.ucl.ac.uk).

FSL-FIRST, version 6.0.1, has previously been described by Patenaude et al. (2011). In short, FSL-FIRST finds the most plausible outline based on the observed intensities from the T1-weighted input image using shape and appearance models derived from a large training dataset. Surface meshes of the subcortical structures were converted to boundary corrected voxelwise segmentations [10].

FreeSurfer, version 6.0.0. is described on the FreeSurferWiki page (https://surfer.nmr.mgh.harvard.edu/fswiki/). In short, labels are assigned to each voxel in the subcortical region (WM + subcortical GM). From these segmentations, the binary segmentations for the individual structure were extracted [11].

Geodesic Information Flow (GIF), versions V2.0, uses manually created atlases for segmentation of the input images. GIF captures the local variation in morphology and in standard space locations. With the use of an iterative geodesic minimization algorithm and the manual labels, more accurate segmentations are expected [12].

VolBrain, version 1.0 is an online pipeline for volumetric brain analysis The proposed pipeline is based on a library of manually labeled atlas cases to perform the segmentation process, including subcortical structure segmentation as proposed by Coupé et al. 2011 [13, 17].

### Brain volume

The normalized brain volume (NBV) and brain volume (BV) were measured with SIENAX (part of FSL version 5.0) [18] on the lesion filled data. SIENAX is the cross-sectional pipeline of the SIENA method [19]. Based on voxel intensities it estimates partial volume fractions of GM, WM and cerebrospinal fluid (CSF) for each voxel. Volumes of GM and WM were added to obtain BV. SIENAX performs normalization of skull size to MNI space to obtain NBV.

### Relation with MS pathology

**Fig. 1:**
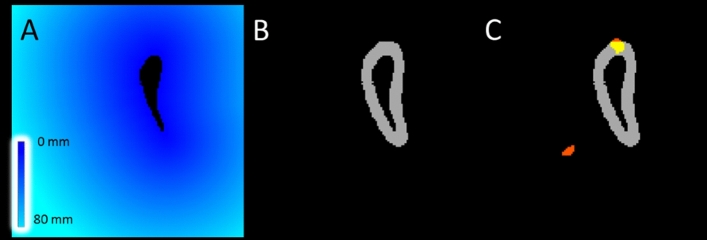
Method for lesion load calculation within a set border. **a** a distance field is created around DGM structure, in this case the caudate nucleus. **b** Distance is set around the DGM structure, seen in grey. **c** An overlay is created between the DGM border and the lesion mask in T1 space. In grey the lesion border is shown, in red the lesions without overlap with the border and in yellow the lesions with an overlap in the border

The association of automatic segmentation performance, as measured by Dice similarity coefficient (DSC, see statistical analyses section), with multiple MS-related disease parameters, i.e. WM lesion load, regional lesion load, normalized brain volume (NBV) and DGM structure volume, was investigated. WM lesion load was determined from the manual lesion outlines. Regional lesion load was evaluated by measuring the lesion load within a pre-defined distance from the DGM structure (see Fig. 1). Using the distance transform, the distance of each voxel to the reference of the structure in the specific subject under investigation was calculated. By thresholding of the subject- and structure-specific distance map and masking with the subject-specific WM mask obtained with FreeSurfer, for each case, a “surrounding WM border” region at distances of 0–10 mm was defined. Region-specific lesion volumes were obtained by masking the WM lesion mask in 3D-T1 space with these WM surrounding WM border.

### Statistical analyses

Intra-rater agreement was measured between the first and second manual segmentation of the structures with the Dice similarity coefficient (DSC) [20]:

$$DSC(A,B) = \frac{2(A \cap B)}{{A + B}}$$

with *A* and *B* defined as the segmentations and where ∩ is the intersection of the two segmentations.

Volumetric analysis was done by comparing the volume of the reference with volumes of the automated segmentation. Moreover, the intra-class correlation coefficient (ICC) for the absolute agreement was calculated [21].

Spatial overlap between reference and the automated method was measured with DSC. Student’s t test was used to compare DSC with the reference between controls and patients. To assess the effect of lesion-filling, two-way ANOVA analysis was performed comparing DSC of native images separately with those from each of the two lesion-filling methods (LEAP and LST).

The relation of disease pathology with spatial performance was examined using Spearman's correlation coefficient, in which 0.0 <|*r*|< 0.2 was considered a weak correlation, 0.2 ≤|*r*|< 0.5 a moderate correlation and |*r*|≥ 0.5 a strong correlation [22].

For all statistical analysis *P*-values < 0.05 were considered statistically significant.

## Results

### Manual segmentation

Volumes of the reference DGM structures are listed in Table 2. Intra-rater agreement was assessed through DSC means per structure for three cases (Table 3). Intra-rater DSC was consistently high, with DSC ≥ 0.85 for all the experts across all six DGM structures. No difference in inter-rater DSC was observed between raters or between DGM structures. The manual labels were combined by majority voting to create the reference segmentation. An evaluation at the voxel level showed similar numbers of voxels segmented by only a single rater in both MS and controls, indicating that there was no greater disagreement between the raters for MS patients compared to controls, see Table 4.

**Table 2:** For all structures and hemispheres, first the mean volume ± standard deviation (std) in millimeter of reference and the four automated segmentation software

Method	Left Caudate			Right Caudate		
*N* = 32	Volume	ICC	DSC	Volume	ICC	DSC
Reference	3.99 ± 0.64			3.98 ± 0.60		
FSL-FIRST	3.46 ± 0.45	0.69	0.84 ± 0.04	3.53 ± 0.49	0.81	0.84 ± 0.04
FreeSurfer	3.55 ± 0.52	0.74	0.76 ± 0.09	3.75 ± 0.58	0.68	0.77 ± 0.09
GIF	3.52 ± 0.42	0.50	0.83 ± 0.04	3.73 ± 0.47	0.60	0.83 ± 0.05
volBrain	3.55 ± 0.55	0.85	0.83 ± 0.07	3.57 ± 0.54	0.86	0.83 ± 0.07

	Left Putamen			Right Thalamus		
	Volume	ICC	DSC	Volume	ICC	DSC
Reference	4.79 ± 0.79			4.65 ± 0.73		
FSL-FIRST	4.85 ± 0.73	0.94	0.88 ± 0.03	4.83 ± 0.79	0.93	0.87 ± 0.04
FreeSurfer	4.60 ± 0.88	0.94	0.81 ± 0.08	4.66 ± 0.88	0.96	0.81 ± 0.07
GIF	4.37 ± 0.70	0.98	0.80 ± 0.03	4.34 ± 0.65	0.92	0.81 ± 0.03
volBrain	4.09 ± 0.65	0.77	0.84 ± 0.06	4.04 ± 0.60	0.80	0.84 ± 0.06

	Left Thalamus			Right Thalamus		
	Volume	ICC	DSC	Volume	ICC	DSC
Reference	6.83 ± 1.19			6.80 ± 1.21		
FSL-FIRST	7.87 ± 1.01	0.76	0.82 ± 0.05	7.69 ± 0.98	0.79	0.83 ± 0.05
FreeSurfer	7.32 ± 1.10	0.80	0.77 ± 0.08	6.93 ± 1.01	0.95	0.78 ± 0.08
GIF	5.81 ± 0.76	0.72	0.77 ± 0.04	5.67 ± 0.68	0.64	0.76 ± 0.04
volBrain	5.47 ± 1.01	0.68	0.80 ± 0.07	5.39 ± 0.98	0.67	0.80 ± 0.07

Second, the intra-class correlation coefficient (ICC) and mean dice similarity coefficient (DSC) ± std between the reference and the segmentation of the automated segmentation software. *N* = amount of subjects

**Table 3:** For all structures and hemispheres the spatial overlap of intra rater agreement. Spatial overlap is shown with the mean dice similarity coefficient ± standard deviation and is calculated over four subjects

Rater	Left caudate	Right caudate	Left putamen	Right putamen	Left thalamus	Right thalamus
Expert 1	0.87 ± 0.031	0.87 ± 0.037	0.89 ± 0.047	0.91 ± 0.026	0.87 ± 0.035	0.88 ± 0.007
Expert 2	0.87 ± 0.051	0.88 ± 0.032	0.85 ± 0.039	0.88 ± 0.018	0.89 ± 0.031	0.88 ± 0.022
Expert 3	0.92 ± 0.004	0.92 ± 0.008	0.91 ± 0.022	0.92 ± 0.016	0.89 ± 0.022	0.91 ± 0.008

**Table 4:** The average (± standard deviation) amount of voxels that were selected by one rater for both healthy control groups (HC) as patients (MS) group

Structure	HC (*n* = 11)	MS (*n* = 21)
Caudate	1356 ± 220	1413 ± 211
Putamen	1460 ± 290	1536 ± 436
Thalamus	2126 ± 443	2083 ± 526

Volumes differed between the Reference and all four automated methods for all structures (all *p* < 0.01), but there were no differences between the volumes for pairs of automated methods

### Performance of automated methods

#### Volumetric agreement

**Fig. 2:**
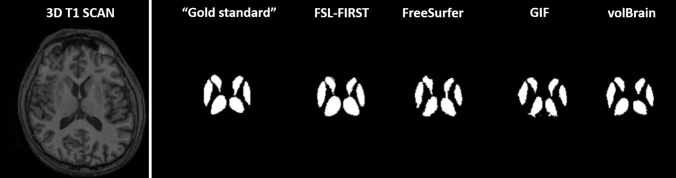
T1 weighted images and segmentation of majority voting, FSL-FIRST, Freesurfer, GIF and volBrain. Segmentations of both left and right hemisphere and for all three structures; caudate, putamen and thalamus

**Fig. 3:**
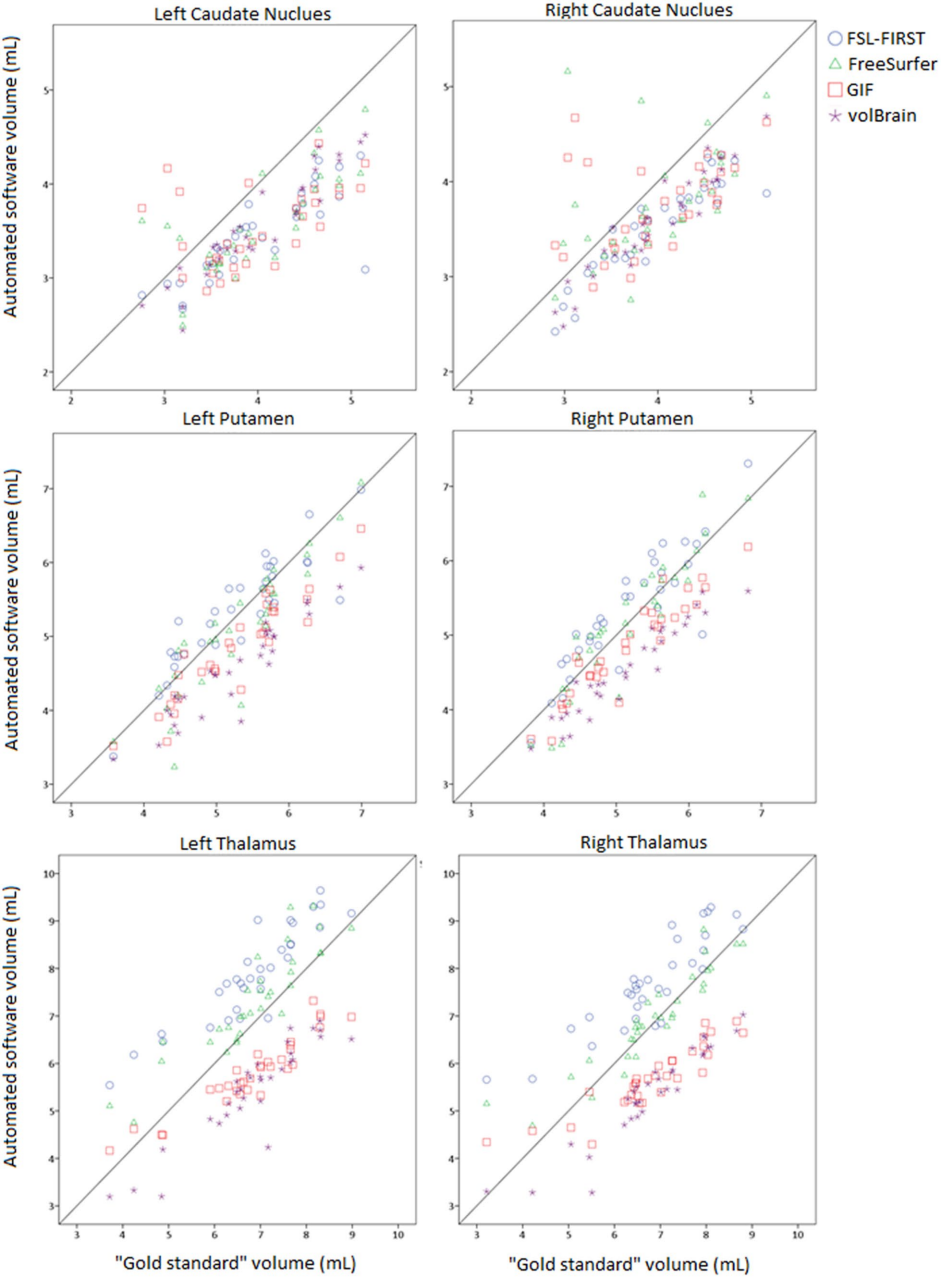
Majority voting segmentation volume and volume by automatic segmentation are given for each deep gray matter structure and segmentation method. Volumes are given in milliliters

DGM volumes of the reference and automatic segmentations were compared. In Fig. 2, an example T1 image is shown along with the corresponding segmentation of reference and automated method. Figure 3 and Table 2 show the volumetric and spatial agreement between reference and automated method. Over the total dataset (*n* = 32) automated average volumes all differed from reference segmentations: caudate and putamen volumes were on average underestimated by all automated method, while thalamus volumes were overestimated by FSL-FIRST and FreeSurfer and underestimated by GIF and volBrain (all *p* < 0.01). Despite these systematic differences, ICC for FSL-FIRST, FreeSurfer and volBrain varied between good (0.60 ≤ ICC < 0.75) and excellent (ICC ≥ 0.75) and for GIF from fair (0.40 ≤ ICC < 0.60) to excellent (Table 3).

### Spatial agreement

**Fig. 4:**
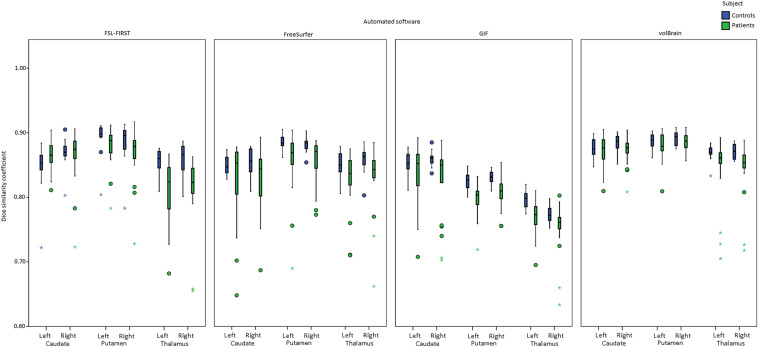
Dice similarity coefficients between segmentations from majority voting and each automated method per DGM structure for both healthy controls (HC) and patients (green)

The DSC between reference and automatic segmentations were assessed. Figure 4, Table 5 show the DSC for both controls and patients. For thalamus, all the DSC were significantly lower for patients compared to controls (*p* < 0.05). For putamen, this was the case for FreeSurfer and GIF, for both left and right hemisphere. The volumes were, however, different in all cases, mostly lower in MS. For the caudate, only a significant difference in DSC between controls and patients was found in the right hemisphere for FreeSurfer and Gif. In all cases, a large variation was observed for patients compared to controls, see Fig. 4.

**Table 5:** For all structures and hemispheres the spatial overlap between the “Gold standard” and the automated segmentation methods for both control and patients group

Method	Caudate nucleus			
	Controls (*N* = 11)		Patients (*N* = 21)	
	Left	Right	Left	Right
FSL-FIRST	0.84 ± 0.44	0.87 ± 0.03	0.86 ± 0.24	0.86 ± 0.04
FreeSurfer	0.85 ± 0.02	0.85 ± 0.02	0.82 ± 0.06	**0.83 ± 0.05**
GIF	0.85 ± 0.02	0.86 ± 0.01	0.83 ± 0.05	**0.83 ± 0.06**
volBrain	0.88 ± 0.02	0.88 ± 0.02	0.87 ± 0.03	0.87 ± 0.02

	Putamen			
	Controls (*N* = 11)		Patients (*N* = 21)	
	Left	Right	Left	Right
FSL-FIRST	0.89 ± 0.03	0.88 ± 0.04	0.88 ± 0.03	0.87 ± 0.04
FreeSurfer	0.89 ± 0.01	0.88 ± 0.01	**0.85 ± 0.05**	**0.86 ± 0.03**
GIF	0.82 ± 0.02	0.83 ± 0.01	**0.80 ± 0.03**	**0.81 ± 0.02**
volBrain	0.89 ± 0.01	0.89 ± 0.01	0.88 ± 0.02	0.89 ± 0.02

	Thalamus			
	Controls (*N* = 11)		Patients (*N* = 21)	
	Left	Right	Left	Right
FSL-FIRST	0.86 ± 0.02	0.86 ± 0.03	**0.81 ± 0.05**	**0.81 ± 0.06**
FreeSurfer	0.85 ± 0.02	0.86 ± 0.02	**0.83 ± 0.05**	**0.83 ± 0.05**
GIF	0.80 ± 0.01	0.77 ± 0.02	**0.77 ± 0.03**	**0.75 ± 0.04**
volBrain	0.87 ± 0.01	0.87 ± 0.01	**0.84 ± 0.05**	**0.84 ± 0.04**

The spatial overlap is given as the mean ± standard deviation of the Dice Similarity Coefficient. Values of patients are bold if they are significantly diferent from those of controls (*p*-value < 0.05). *N* = amount of subjects

### Relation with pathology

**Fig. 5:**
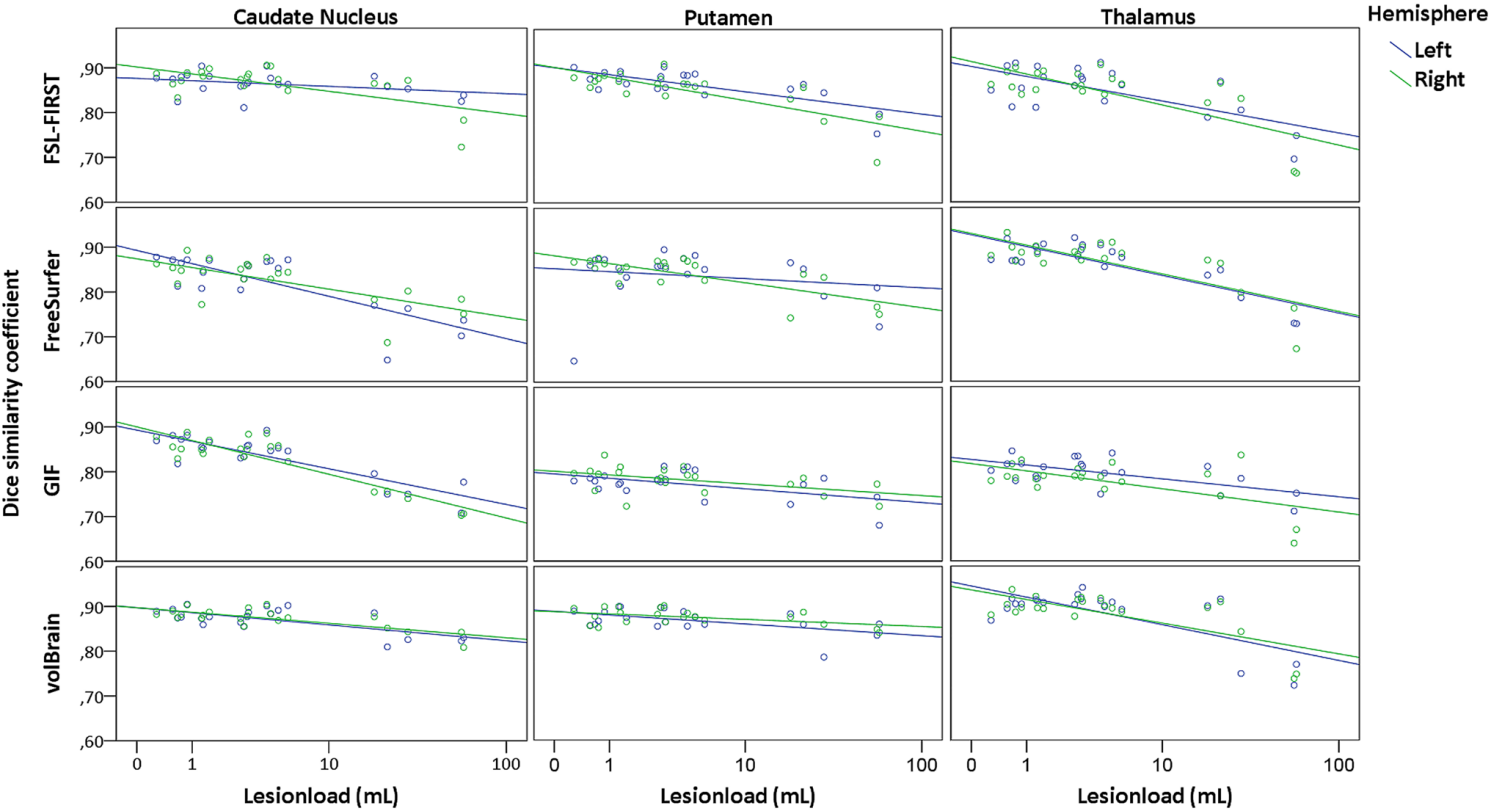
Dice similarity coefficients versus lesion load, represented per DGM structure and segmentation method and left (blue) and right (green) hemisphere

Higher WM lesion load was associated with a lower performance of the automated method: total WM lesion load was negatively correlated with DSC for all method (Table 6 and Fig. 5). The correlation was moderate to strong for all structures and for all methods and both sides (|*r*|> 0.2), however, not all were significant, see Table 6. The regional WM lesion load, i.e., that located within 10 mm of the structure, was also negatively correlated with DSC (Table 6), however, these correlations ranged from weak to moderate for putamen and thalamus and for caudate from moderate too strong.

**Table 6:** Spearman correlation between the dice similarity index and lesion load (LL), regional lesion load (RLL), normalized brain volume (NBV) and volume of region of interest (ROIV)

Method	Left caudate				Right caudate			
N = 21	LL	RLL	NBV	ROIV	LL	RLL	NBV	ROIV
FSL-FIRST	− 0.31	− 0.52*	− 0.88	0.45*	− 0.33	− 0.41	0.36	0.53**
FreeSurfer	− 0.60**	− 0.49*	0.20	0.47**	− 0.57**	− 0.62*	0.25	0.37*
GIF	− 0.68**	− 0.57**	0.25	0.17	− 0.57**	− 0.63*	0.34	0.38*
volBrain	− 0.34	− 0.43	0.17	0.61**	− 0.57**	− 0.58**	0.18	0.82**

	Left Putamen				Right Putamen			
	LL	RLL	NBV	ROIV	LL	RLL	NBV	ROIV
FSL-FIRST	− 0.56**	− 0.30**	0.69**	0.72**	− 0.69**	− 0.80**	0.43	0.62**
FreeSurfer	− 0.26	− 0.45	0.08	0.74**	− 0.56**	− 0.20	0.23	0.37**
GIF	− 0.26	− 0.25	0.52	0.65**	− 0.54*	− 0.50	0.40	0.59**
volBrain	− 0.39	− 0.09	0.43	0.56**	− 0.34	− 0.68**	0.44*	0.65**

	Left Thalamus				Right Thalamus			
	LL	RLL	NBV	ROIV	LL	RLL	NBV	ROIV
FSL-FIRST	− 0.46*	− 0.16	0.36	0.54**	− 0.52*	− 0.27	0.29	0.42*
FreeSurfer	− 0.53*	− 0.30	0.30	0.26	− 0.49*	− 0.38	0.46*	0.64*
GIF	− 0.42	− 0.43	0.18	0.52**	− 0.23	− 0.29	0.18	0.44*
volBrain	− 0.30	− 0.16	0.45*	0.48**	− 0.31	− 0.26	0.41	0.63**

Correlation is measured for all structures, hemispheres and automated segmentation software. With *α* for * < 0.05 and ** < 0.01 for significant spearman correlation. *N* = amount of subjects

Both NBV and the volume of the structure of interest itself were positively correlated with DSC (Table 6). The correlations between NBV and DSC were often not significant and ranged between weak and strong for the different structures, methods and sides. The correlations between the volume of the structure and DSC were often significant. Only the correlation of DSC and volume measured on left caudate with GIF and on left thalamus with FreeSurfer were not significant. The correlations ranged for the putamen, thalamus and right caudate from moderate too strong and for left thalamus from weak too strong.

To overcome the effect of lesions, two lesion-filling methods (LST-LF and LEAP) were used. Two-way ANOVA analysis per hemisphere, per method and DGM structure showed no significant difference in segmentation volume and DSC for both lesion filling methods compared to native (non-filled) patients images (see Supplementary Fig. 1 and Supplementary Table 5). Moreover, Student’s *t* test showed no significant difference in segmentation volume or DSC for either of the filling methods compared to native patient images (Table 7).

**Table 7:** For all structures, hemispheres and automated segmentation method the *p*-value of students *t* test between dice similarity index of non-filled and filled T1 images before applying automated segmentation method

Method	Left Caudate		Right Caudate	
*N* = 21	LEAP	LST-filling	LEAP	LST-filling
FSL-FIRST	0.70	0.90	0.79	0.73
FreeSurfer	0.73	0.75	0.16	0.56
GIF	0.84	0.76	0.78	0.54
volBrain	0.75	0.84	0.84	0.79

	Left Putamen		Right Putamen	
	LEAP	LST-filling	LEAP	LST-filling
FSL-FIRST	0.55	0.92	0.91	0.97
FreeSurfer	0.89	0.56	0.89	0.97
GIF	0.36	0.86	0.25	0.90
volBrain	0.86	0.95	0.79	0.51

	Left Thalamus		Right Thalamus	
	LEAP	LST-filling	LEAP	LST-filling
FSL-FIRST	0.56	0.95	0.63	0.95
FreeSurfer	0.85	0.87	0.86	0.98
GIF	0.84	0.96	0.94	0.88
volBrain	0.98	0.98	0.78	0.89

Filling is done with LEAP and LST

*N* = amount of subjects

## Discussion

Using a systematic and objective evaluation against a consensus of manual segmentations in a multi-center dataset, this study provides evidence that automated DGM segmentation methods performed worse on brain scans of MS patients than on those of healthy controls. Higher lesion volumes were associated with poorer DGM segmentation performance.

The accuracy of DGM segmentations is not an academic question but also has great clinical importance. Clinical and cognitive deterioration in MS have been linked to brain and GM atrophy [5, 23, 24], and several treatments are now available that are able to reduce brain atrophy rates in MS [25–28]. Accurate measurement of the volumes of DGM structures in MS is becoming especially important, because of the strong relation of DGM atrophy with cognitive impairment (1,5). This study reveals that existing DGM segmentation methods perform not as accurate in MS patients as in controls, as reflected by the lower DSC (overlap) scores. This implies that the results may incorporate increased random variability and bias when applied to MS cases and should be interpreted with great caution. In the future, methodological improvements are required to achieve better performance in MS.

Only a limited number of studies directly investigated the performance of DGM segmentation methods when applied to MS. Derakhshan et al. (2010) evaluated six automated segmentation method for GM atrophy on T1 MR images of three MS patients. They concluded that severe shortcomings are present in the segmentation of DGM structures [1]. The current study extends those findings substantially by investigating a multi-center dataset comprising 21 MS subjects and 11 controls using full three-dimensional manual segmentations of three DGM structures bilaterally. Importantly, using this dataset we were able to objectively compare several widely applied automated segmentation techniques in a multi-center setting. By selecting from previously acquired data a subset that maximized the number of scanners and the number of progressive patients, we were able to demonstrate quantitatively that this performance impairment exists in MS patients with a relatively long disease duration and/or progressive course. It would next be important to confirm this independently, as well as to investigate if the effect already occurs in early MS or CIS, given that DGM atrophy already occurs at those early stages [29].

To obtain insights that could aid in amending the impairment of segmentation performance, we investigated several possible causes. One important candidate reason for the reduced accuracy of DGM segmentation in MS is formed by the focal WM lesions. Previous work on whole-brain total GM volume measurement has shown that MS WM lesions affect the GM volume measurement for a number of different packages [6–9, 30]. Similarly, the presence of local or overall brain atrophy or diffusions damage could affect the performance of segmentation methods [31]. The precise mechanism behind these deteriorating effects may differ between packages but could include effects on image intensity histograms, image registration and non-brain tissue removal [5]. Therefore, we investigated whether total lesion load, regional lesion load, NBV and the volume of the structure itself were related to the performance of the automated DGM segmentation method. The strongest association with poorer accuracy in MS cases compared to healthy controls was observed for total WM lesion load. Higher regional lesions load and lower total and local brain volumes were also associated with poorer performance, but less strongly. It should be mentioned that the small size of regional lesion load—which is confined to a narrow region around the structure of interest—and the relatively small number of MS patients may have hampered our ability to detect this association. Moreover, we should mention that the relation between the volume of the structure and the performance of the automated DGM segmentation methods could also result from the artifact that the performance is dependent on the volume (greater volume could result in higher DSC). Therefore, the positive relationship should be studied in more detail for a better understanding of this effect.

While the accuracy of the segmentation was the most important focus of the present work, the accuracy of the resulting volumes may be considered at least equally important from a clinical viewpoint. Here to, systematic differences were observed between the automated methods and the reference measurements. We also saw a difference between the volumes obtained from different methods for each structure separately. The automated methods underestimated volumes of caudate and putamen while the volume of the thalamus was generally overestimated. This difference could be related to the different anatomical definitions used in the manual standard and the automated methods. One specific example is the question of whether the lateral geniculate nuclei bodies should be included or excluded when segmenting the thalamus [32]. The differences for the structures could also be indirectly related to disease effects: due to the anatomical location of the structures, some brain regions are more prone to contain lesions than others or could be more affected by regional atrophy (both of which could impair DGM segmentation). A study with more patients and a more diverse lesion load could give more insight if the automated method performs differently on DGM structures. Moreover, a study on spatial patterns on the DGM structures could also give more insight into the performance of the methods.

It has been suggested that filling lesions increases the accuracy of total GM segmentation, and we also expected an improvement of DGM segmentation after lesions filling [6, 30, 33]. However, we measured no difference in the performance of the automated method compared to manual segmentation after filling lesions. This is similar to the results reported in 2014 by Popescu et al. for filling with FLS-lesion filling and LEAP and segmentation with FSL-First for multiple DGM structures (e.g. thalamus, putamen, caudate nucleus, brainstem) (7). Therefore, it seems that lesion filling increases the accuracy of total GM segmentation, however, it does not increase the accuracy of DGM segmentation. Our hypothesis on this is that an underlying factor such as regional atrophy or GM lesion load or a combination of the pathology aspects (e.g. lesion load, atrophy, NAWM, diffusion damage) could be a cause. It should be mentioned that the lesions were manually outlined on either T2/PD images or FLAIR images, potentially leading to differences in the lesion segmentations that could have affected our results. However, after dividing the group into two different sets the same effects were visible as in the complete group, though less significant, as expected for the smaller group sizes.

Moreover, we suggest a study on the effect of WM-GM contrast-to-noise ratio which might cause this effect. As MS pathology affects both the WM and GM, resulting in more variation in the WM and GM signal, it is possible that the WM-GM contrast ratio is changed. Ratio could be changed due to iron change or damage of WM and/or GM [34]. Westlye et al. (2009) showed in Alzheimer’s Disease (AD) that cortical thickness in subjects with regionally reduced tissue contrast was overestimated compared to subjects without reduces tissue contrast. They indicate that the overestimation is related to alterations in myelin density and water compartment close to the WM. Moreover, adjusting for local variability in tissue contrast could correct the overestimation [35]. Therefore, further studies should investigate this in MS and, moreover, other possible causes (e.g. diffuse signal changes, the effect of image processing) should be investigated as well.

Furthermore, as it is important to have segmentation with accurate spatial level for correct localization and shape, future research could take are more in-depth approach regarding shape analysis e.g. quantitative vertex displacement analysis [36]. This analysis enables the finding of vertices which have a significantly different shape from the reference and would be of added value in unraveling why some packages are outperforming others.

In conclusion, the performance of four state-of-the-art automated DGM segmentation method is impaired in MS, which warrants caution in interpreting DGM volumes both in group studies and in individual patients. Poorer accuracy was associated with higher WM lesion load and smaller global-local brain volumes, but the mechanism is not yet understood. Remarkably, the impaired performance was not improved by lesion-filling. More research is needed to understand the underlying causes of reduced accuracy and then eliminate their effects.

## Electronic supplementary material

Below is the link to the electronic supplementary material.

Supplementary file1 (PNG 154 kb)

Supplementary file2 (DOCX 181 kb)

Supplementary file3 (PNG 100 kb)

## Data Availability

Data are not available for other research groups, because of ethical and privacy issues.
