# Supplementary figures and images for: Reduced accuracy of MRI deep grey matter segmentation in multiple sclerosis: an evaluation of four automated methods against manual reference segmentations in a multi-center cohort
Source: J Neurol. 2020 Jul 3;267(12):3541–54. doi: 10.1007/s00415-020-10023-1 (PMC7674567; doi:10.1007/s00415-020-10023-1)

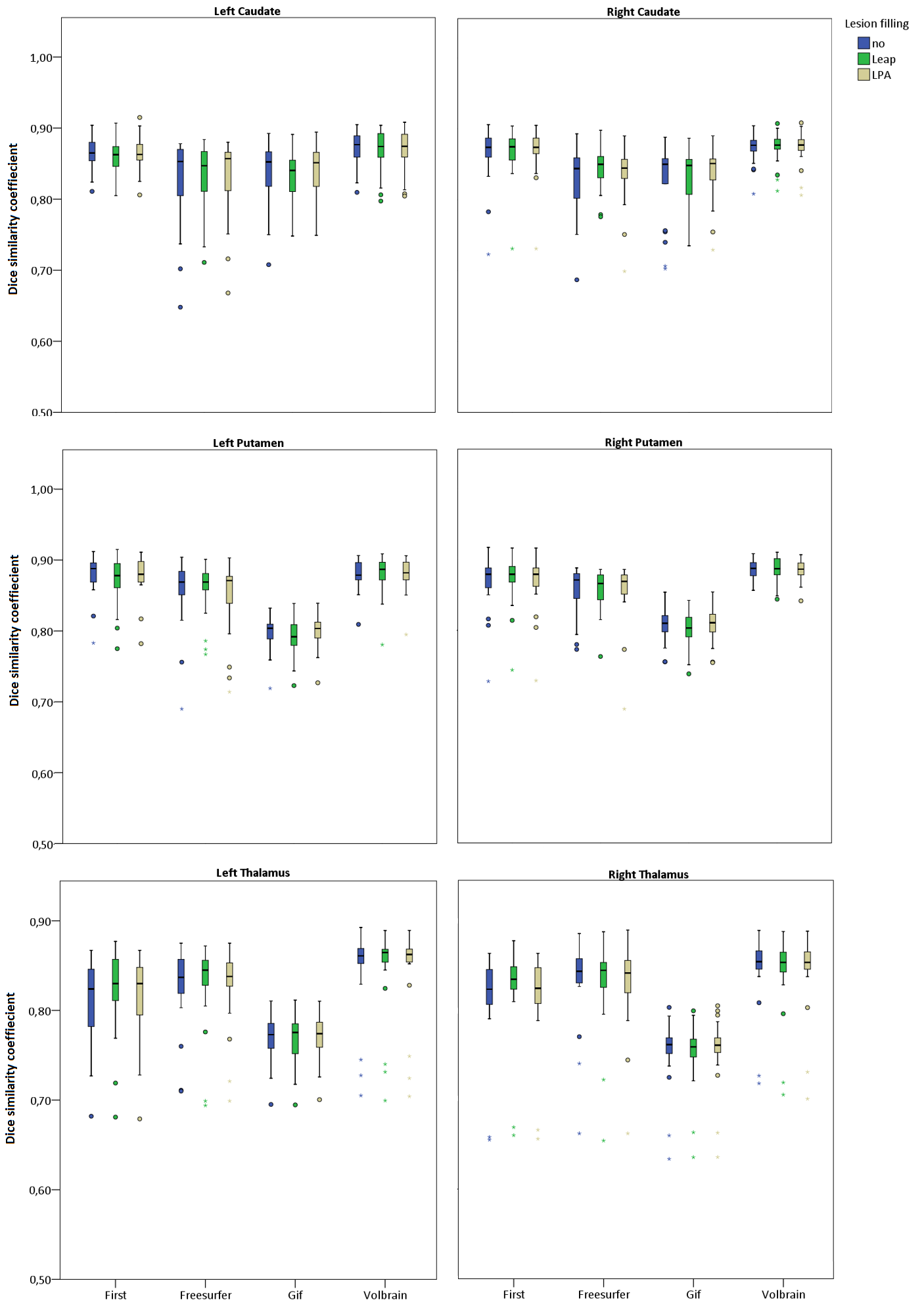

Supplement: Supplementary file 1 — Supplementary file1 (PNG 154 kb) [file 415_2020_10023_MOESM1_ESM.png]

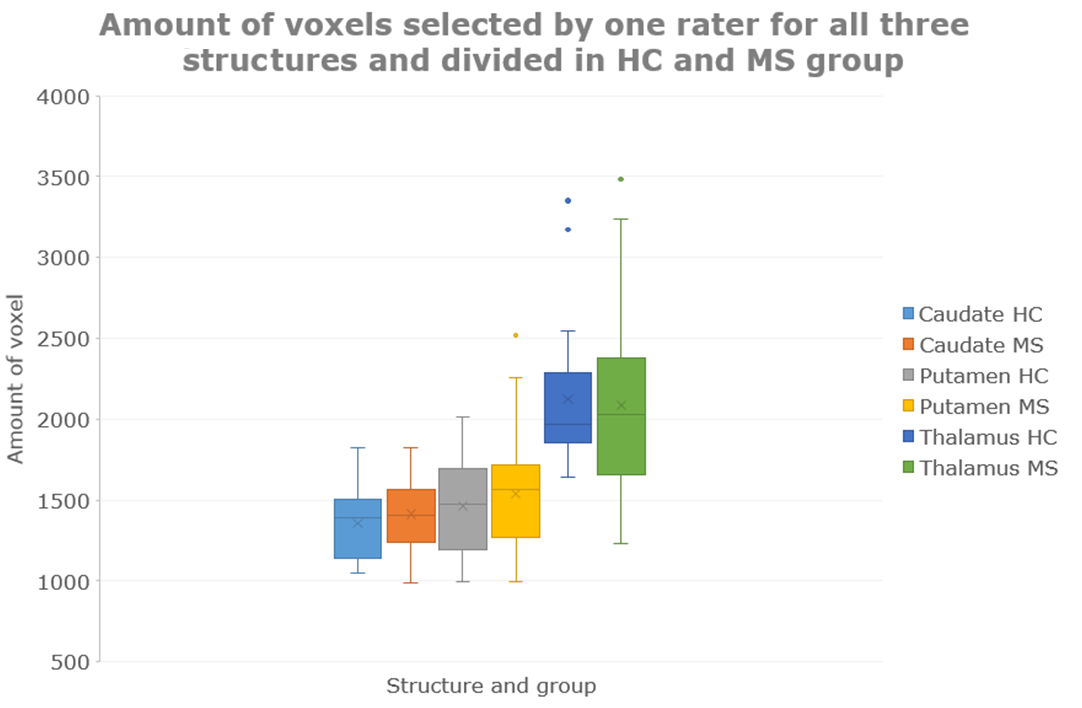

Supplement: Supplementary file 3 — Supplementary file3 (PNG 100 kb) [file 415_2020_10023_MOESM3_ESM.png]
