# Supplementary material for: Reduced accuracy of MRI deep grey matter segmentation in multiple sclerosis: an evaluation of four automated methods against manual reference segmentations in a multi-center cohort
Source: J Neurol. 2020 Jul 3;267(12):3541–54. doi: 10.1007/s00415-020-10023-1 (PMC7674567; doi:10.1007/s00415-020-10023-1)
Supplement: Supplementary file 2 — Supplementary file2 (DOCX 181 kb) [file 415_2020_10023_MOESM2_ESM.docx]

**Supplementary data A:**

The nine centers which recruited subjects for this study:

1 Amsterdam UMC – location VUmc, Amsterdam (The Netherlands)

2 Hospital Vall d’Hebron, Barcelona (Spain)

3 University Hospital Basel, Basel, (Switzerland)

4 Glostrup University Hospital, Cophagen, (Denmark)

5 Medical University Graz, Graz (Austria)

6 University College London, London (UK)

7 San Raffaele Scientific Institute, Milan (Italy)

8 Second University of Naples, Naples (Italy)

9 University of Siena, Siena (Italy).

**Supplementary data B:**

Description of LST-LF and LEAP

LST-lesion filling (LST-LF) (Reference 16 of the main paper; Schmidt P. et al., Neuroimage 59(4):3774–3783) requires a lesion mask together with a 3D T1 weighted image and FLAIR image as input. LST-lesion filling is part of the Lesion Segmentation Toolbox (LST). For the filling part, also the lesion segmentation part of the LST methods is needed. Therefore, we first segmented lesions with LST, and afterwards replaced the generated lesion mask with the manually outlined lesion mask. This ensures that the LST-lesion filling will fill the same lesions as the other methods. LST-lesion filling uses local information, which allows accurate filling of lesions even on images that are not bias-field corrected.

LEAP extracts the brain. Next the images are corrected for non-uniformity using N3 (Reference 8 of the main paper, Chard DT et al., J Magn Reson Imaging. 32(1):223–228). A 3D image containing WM signal intensity values is generated. without any lesions. based on the noise and signal inhomogeneity of the original image. The last step in the LEAP pipeline is to replace the lesion intensities in the original image with intensity values taken from corresponding locations in the simulated WM image.

**Supplementary Protocol**

A protocol for the manual segmentation of the thalamus, caudate and putamen using high resolution MRI.

J. Burggraaff^1*^, Y. Liu^2*^, J. Simoes^2*^, M.P. Wattjes^2^, F. Barkhof^2^, C.R.G. Guttmann^3^, H. Vrenken^2^

^*^Equally contributing authors

Affiliation

^1^ Department of Neurology, Amsterdam UMC – location VUmc (Amsterdam, NL)

^2^ Radiology and Nuclear Medicine, Amsterdam UMC – location VUmc (Amsterdam, NL)

^3^ Center for Neurological Imaging, Department of radiology, Brighman and Women’s Hospital, Harvard Medical School (Boston, Massachusetts, USA)

This is a condensed version of the segmentation protocol used in the study. The full segmentation protocol describes how to manually trace, on 3D T1-weighted MRI-scans, three deep grey matter (DGM) structures: the thalamus, caudate, and putamen. The protocol describes the procedure of the manual segmentation, the anatomical borders of the structures and how the edges of the structures can be traced. The segmentation procedure consists of two phases: first, demarcating the edges of the DGM structures on orthogonal scans, and second, outline and filling the inside of the path the rater has defined by the borders of the structures in axial view.

The borders of each structure are defined as follows:

*Caudate nucleus:*

The anterior border is separated by fibers of the anterior limb of the internal capsule.

The posterior border is the WM and no structures that may interfere with its identification.

The inferior border is directly above the accumbus nucleus.

The superior border lies above the thalamus and lateral to the lateral ventricle.

The medial border is the lateral ventricle.

The lateral border is the WM and no structures that may interfere with its identification

*Putamen:*

The anterior border is bounded by the anterior limb of the internal capsule and the anterior perforated substance.

The posterior border is separated from the caudate by parts of the anterior and posterior limb of the internal capsule.

The inferior border is the external capsule.

The superior border is the internal capsule.

The medial border is located the external medullary lamina of the globus pallidus, pallidum or internal capsule.

The lateral border is the small amount of WM between the lateral part of the putamen and claustrum.

*Thalamus:*

The anterior border is just posterior to the commissure.

The posterior border is the superior and inferior colliculi and the atrium of the lateral ventricle and/or the tail of the hippocampus.

The superior border is the lateral ventricle and the fornix.

The inferior border is the zona incerta and it junction with the internal capsule.

The medial border is the third ventricle.

The lateral border is the medial border of both the genu and the posterior limb of the internal capsule.

| **Center** | **TR (ms)** | **TE (ms)** | **TI (ms)** | **FA (◦)** | **Acquisition (Voxel size (mm3))** |
| --- | --- | --- | --- | --- | --- |
| A | 7.8 | 3 | 450 | 12 | 256x256x188 (0.976x0.976x1) |
| B | 2300 | 2.98 | 900 | 9 | 232x256x176 (1x1x1) |
| C | 1570 | 2.70 | 900 | 9 | 160x256x256 (1x1x1) |
| D | 6.9 | 2.78 | 831 | 9 | 160x240x240 (1x1x1) |
| E | 1900 | 2.1 | 900 | 9 | 224x256x176 (1x1x1) |
| F | 2200 | 2.94 | 900 | 10 | 256x192x192 (1x1x1) |
| G | 8.3 | 3.72 | 1000 | 8 | 256x256x192 (1x1x1) |
| H | 5.5 | 1.76 | 450 | 10 | 256x256x188 (1x1x1) |
| I | 8.3 | 3.72 | 1000 | 8 | 256x256x192 (1x1x1) |
| **Supplementary Table 1: Acquisition parameter of T1 image overview per center. Abbreviations: TR=repetition time, TE=echo time, TI=inversion time, FA=flip angle.** | | | | | |

| **Method** | **Left Caudate**  **p -value ANOVA <0.001** | | | | **Right Caudate**  **p -value ANOVA <0.001** | | | |
| --- | --- | --- | --- | --- | --- | --- | --- | --- |
|  | FSL-FIRST | FreeSurfer | GIF | volBrain | FSL-FIRST | FreeSurfer | GIF | volBrain |
| FSL-FIRST | - | <0.001 | 0.004 | <0.001 | - | <0.001 | <0.001 | <0.001 |
| FreeSurfer | <0.001 | - | <0.001 | <0.001 | <0.001 | - | <0.001 | <0.001 |
| GIF | 0.004 | <0.001 | - | <0.001 | <0.001 | <0.001 | - | <0.001 |
| volBrain | <0.001 | <0.001 | <0.001 | - | <0.001 | <0.001 | <0.001 | - |
|  | **Left Putamen**  **p -value ANOVA <0.001** | | | | **Right Putamen**  **p -value ANOVA <0.001** | | | |
|  | FSL-FIRST | FreeSurfer | GIF | volBrain | FSL-FIRST | FreeSurfer | GIF | volBrain |
| FSL-FIRST | - | <0.001 | <0.001 | 0.569 | - | <0.001 | <0.001 | 0.002 |
| FreeSurfer | <0.001 | - | 0.723 | <0.001 | <0.001 | - | 0.433 | <0.001 |
| GIF | <0.001 | 0.723 | - | <0.001 | <0.001 | 0.443 | - | <0.001 |
| volBrain | 0.569 | <0.001 | <0.001 | - | 0.002 | <0.001 | <0.001 | - |
|  | **Left Thalamus**  **p -value ANOVA <0.001** | | | | **Right Thalamus**  **p -value ANOVA <0.001** | | | |
|  | FSL-FIRST | FreeSurfer | GIF | volBrain | FSL-FIRST | FreeSurfer | GIF | volBrain |
| FSL-FIRST | - | <0.001 | <0.001 | <0.001 | - | <0.001 | <0.001 | <0.001 |
| FreeSurfer | <0.001 | - | 0.733 | <0.001 | <0.001 | - | 0.016 | <0.001 |
| GIF | <0.001 | 0.733 | - | <0.001 | <0.001 | 0.016 | - | <0.001 |
| volBrain | <0.001 | <0.001 | <0.001 | - | <0.001 | <0.001 | <0.001 | - |
| **Supplementary Table 3: p-value of ANOVA repeated measurement of Dice similarity coefficient and post hoc analysis between the software for all hemispheres and structures.** | | | | | | | | |

| **Method** | **Left Caudate** | | **Right Caudate** | |
| --- | --- | --- | --- | --- |
|  | Volume | DSC | Volume | DSC |
| F-value | 0.030 | 0.111 | 0.771 | 0.566 |
| P-value | 0.99 | 0.90* | 0.46 | 0.57 |
|  | **Left Putamen** | | **Right Putamen** | |
|  | Volume | DSC | Volume | DSC |
| F-value | 0.379 | 0.194 | 0.003 | 0.049 |
| P-value | 0.69 | 0.82 | 0.99 | 0.95 |
|  | **Left Thalamus** | | **Right Thalamus** | |
|  | Volume | DSC | Volume | DSC |
| F-value | 0.003 | 0.044 | 0.422 | 0.008 |
| P-value | 0.99 | 0.96 | 0.66 | 0.99 |
| **Supplementary Table 4: ANOVA repeated measurement between non filling and filling with both LEAP and LST** | | | | |


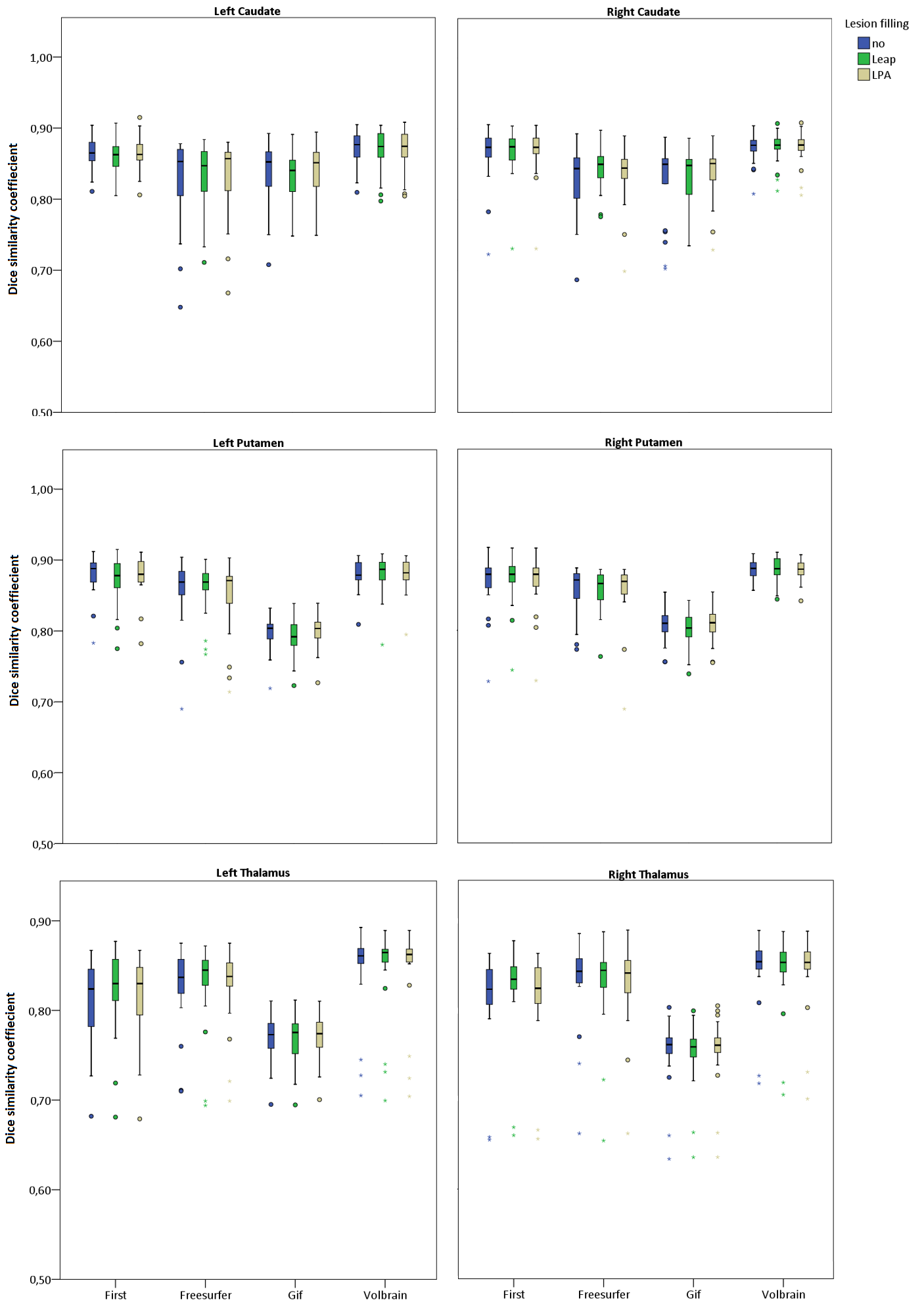


Supplementary Figure 1: Dice similarity coefficient of automated methods with manual reference for all structures, hemispheres and unfilled lesions and filled lesions.
